# "Canvassing Will Disqualify" (?)

**Published:** 1914-12-26

**Authors:** 


### THE EDITOR'S LETTER-BOX.

### "Canvassing Will Disqualify" (?).

*To the Editor of* The Hospital.

Sir,—The following personal experience appears to me to support your recent argument that, though canvassing is punishable by disqualification, the penalty is not always enforced, while disregard of the warning possesses advantages.

About eight weeks ago an advertisement appeared in the *Lancet* notifying some vacancies in the staff of lecturers on hygiene, nursing, and the care of children employed by the London County Council. Having more evening leisure just now than is usual, or indeed acceptable to me, I applied for a form (on which was printed in bold type that canvassing would disqualify), filled it in, and awaited results. I am a practitioner of nearly thirty years' experience; have held the appointment of medical officer of health; have in earlier years taken pupils in general education, as well as medicine; have had some experience of lecturing; and am the author of works on various literary subjects. I declared myself willing to lecture on hygiene, nursing, etc., any night, in any week, and in any London district. But I did not canvass, and my application remains unanswered. Unless canvassing was employed, each of the selected candidates possessed qualifications superior to mine. I wonder.—Yours faithfully,

Persicus.

